# Elucidating the biological basis for the reinforcing actions of alcohol in the mesolimbic dopamine system: the role of active metabolites of alcohol

**DOI:** 10.3389/fnbeh.2013.00104

**Published:** 2013-08-23

**Authors:** Gerald A. Deehan, Sheketha R. Hauser, Jessica A. Wilden, William A. Truitt, Zachary A. Rodd

**Affiliations:** Department of Psychiatry, Institute of Psychiatric Research, Indiana University, School of MedicineIndianapolis, IN, USA

**Keywords:** acetaldehyde, salsolinol, ethanol, reinforcement (psychology), reward, dopamine

## Abstract

The development of successful pharmacotherapeutics for the treatment of alcoholism is predicated upon understanding the biological action of alcohol. A limitation of the alcohol research field has been examining the effects of alcohol only and ignoring the multiple biological active metabolites of alcohol. The concept that alcohol is a “pro-drug” is not new. Alcohol is readily metabolized to acetaldehyde within the brain. Acetaldehyde is a highly reactive compound that forms a number of condensation products, including salsolinol and iso-salsolinol (acetaldehyde and dopamine). Recent experiments have established that numerous metabolites of alcohol have direct CNS action, and could, in part or whole, mediate the reinforcing actions of alcohol within the mesolimbic dopamine system. The mesolimbic dopamine system originates in the ventral tegmental area (VTA) and projects to forebrain regions that include the nucleus accumbens (Acb) and the medial prefrontal cortex (mPFC) and is thought to be the neurocircuitry governing the rewarding properties of drugs of abuse. Within this neurocircuitry there is convincing evidence that; (1) biologically active metabolites of alcohol can directly or indirectly increase the activity of VTA dopamine neurons, (2) alcohol and alcohol metabolites are reinforcing within the mesolimbic dopamine system, (3) inhibiting the alcohol metabolic pathway inhibits the biological consequences of alcohol exposure, (4) alcohol consumption can be reduced by inhibiting/attenuating the alcohol metabolic pathway in the mesolimbic dopamine system, (5) alcohol metabolites can alter neurochemical levels within the mesolimbic dopamine system, and (6) alcohol interacts with alcohol metabolites to enhance the actions of both compounds. The data indicate that there is a positive relationship between alcohol and alcohol metabolites in regulating the biological consequences of consuming alcohol and the potential of alcohol use escalating to alcoholism.

## Introduction

Alcoholism and alcohol (EtOH) abuse is a global burden. Alcoholism is estimated to be responsible for 3.8% of all global deaths, and cost associated with treatment equivalent to 1% of gross national product of high- and medium-income countries (Rehm et al., 2009). As such, a great deal of research has focused on therapeutic interventions to aid individuals that are currently suffering from alcoholism and a great deal of effort has been put forth to identify neurobiological traits that are common in individuals that are at a high-risk to develop an alcohol-use disorder. However, while several lines of research have emerged focusing on the many different facets of EtOH addiction the biological basis of the reinforcing properties of EtOH has not been completely established. Opposing theories have emerged with some suggesting that it is the action of the EtOH molecule itself that underlies the rewarding properties of EtOH. Others believe that EtOH is simply a “pro-drug” and the rewarding properties of EtOH are dependent on the action of the metabolites/byproducts of EtOH within the brain. The main principles underlying the “pro-drug” theory assert that (1) following EtOH consumption, EtOH concentrations within the body are unable to reach levels that adequately affect the central nervous system (CNS), (2) various behavioral and physiological effects of EtOH endure well past the bioavailability of EtOH in the system, and (3) manipulation of the metabolism of EtOH, and the subsequent formation of the metabolites and/or byproducts, within the system affects most, if not all, of the CNS effects of EtOH. The contrary theory suggests that EtOH affects several neurotransmitter systems thereby exerting its effects within the CNS. Proponents of this theory suggest there is no conclusive evidence that the metabolites of EtOH possess the ability to cross the blood brain barrier and the metabolites exists for too short a period to mediate the more persistent effects of EtOH intoxication. Regardless of such polarized stances, EtOH reward within the CNS likely depends on the action of EtOH in conjunction with its metabolites/byproducts. This review will present an overview of the behavioral and neurochemical actions of the neuroactive metabolite acetaldehyde (ACD), and subsequent metabolites/byproducts (i.e., salsolinol) formed through the reaction/condensation of ACD with endogenous compounds, within the central and peripheral nervous systems following EtOH intake.

## The first metabolite of alcohol: acetaldehyde

It has been well established that high levels of ACD within the periphery are associated with aversive symptoms (i.e., flushing, headaches, etc.). The drug disulfuram (tetraethylthiuramdisulphide), which has been approved for the treatment of alcoholism, exacerbates the aversive symptoms of ACD by inhibiting the metabolism of ACD thereby encouraging individuals to abstain from EtOH consumption. The mechanisms of action behind disulfuram treatment were discovered serendipitously. In the early 20th century, a report emerged describing individuals who worked in a metal manufacturing plant experiencing transitory aversive symptoms (i.e., fatigue, shortness of breath, flushing of the face, increased heart rate, headaches) following the consumption of alcoholic beverages (Koelsch, 1914). Such symptoms were subsequently linked to the compound calcium cyanamide, an organic compound used in the production of metals, which the workers were in regular contact with. Similar symptoms were reported shortly thereafter in patients that had consumed ink cap mushrooms prior to drinking EtOH; a reaction that was linked to the amino acid coprine present in the mushroom (Chifflot, 1916; Reynolds and Lowe, 1965). Two decades later, Williams (1937) suggested that the cure of alcoholism may have been discovered as workers at a rubber plant that were exposed to the compound tetramethylthiuram experienced similar aversive symptoms to those outlined above when they consumed EtOH.

Soon thereafter, two researchers, Erik Jacobsen and Jens Hald, began examining tetraethylthiuramdisulphide (disulfuram) as a possible treatment for intestinal worms. Utilizing themselves as test subjects, both men reported experiencing several aversive symptoms following EtOH consumption (i.e., sleepiness, increased heart rate, etc.; Jacobsen, 1958). Follow-up studies indicated that disulfuram, since marketed as antabuse, acted to block aldehyde dehydrogenase, an enzyme that metabolizes ACD, causing increased blood ACD levels thereby increasing the aversive side effects of EtOH consumption (Hald and Jacobsen, 1948). Early studies had already indicated a positive correlation between EtOH intake and increased blood ACD levels such that binge drinkers exhibited ACD levels 35 times greater than controls (Stotz, 1943). However, additional studies indicated that social drinkers treated with antabuse exhibited blood ACD levels 5–10 times greater than individuals that did not receive the treatment (Hald and Jacobsen, 1948; Larsen, 1948). Treating individuals with antabuse, prior to EtOH consumption, allowed for the detection of ACD in the breath (Hald and Jacobsen, 1948). Preclinical research indicated that antabuse rendered ACD detectable in the breath of rabbits following EtOH exposure and research aimed at the identification of the metabolic pathway of EtOH began (Hald et al., 1949a,b).

Over the next 30 years, several theories emerged as to the function of ACD in EtOH-use disorders (Carpenter and Macleod, 1952; Myers and Veale, 1969; Davis and Walsh, 1970; Truitt and Walsh, 1971; Griffiths et al., 1974). A number of theories identified EtOH as a “pro-drug” suggesting alcoholism would be better termed “acetaldhydeism” as ACD was responsible for all of the effects associated with the imbuement of EtOH (Truitt and Walsh, 1971; Raskin, 1975). Contradictory theories asserted that ACD in no way mediated the effects of EtOH. Such assertions were supported by research showing that the consumption of EtOH produced only trace levels of ACD in the cerebrospinal fluid and brain (Kiianmaa and Virtanen, 1978; Pikkarainen et al., 1979; Eriksson et al., 1980) and that ACD was unable to cross the blood brain barrier except when in exceedingly high concentrations (Sippel, 1974; Tabakoff et al., 1976; Eriksson, 1977; Petersen and Tabakoff, 1979). However, Cohen et al. (1980) reported that the local formation of ACD within the brain was possible thereby reestablishing the ACD/EtOH debate.

## Acetaldehyde and alcoholism: a genetic perspective

**Figure 1:**
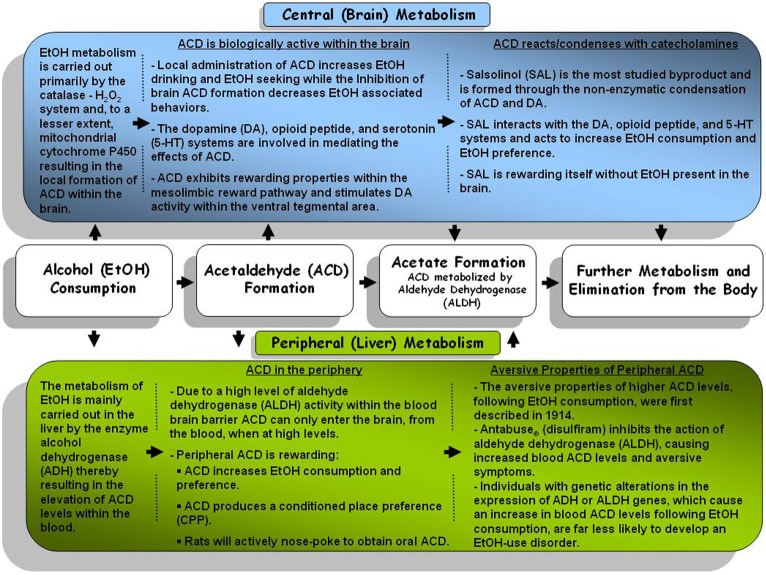
**A general schematic representation of the central (brain) and peripheral (body) metabolism pathways for alcohol and alcohol metabolites**.

Following consumption, EtOH undergoes a number of reactions as it is metabolized. The primary pathway through which EtOH is eliminated from the body involves the action of the alcohol dehydrogenase (ADH) and aldehyde dehydrogenase (ALDH) enzymes (for review see: Deehan et al., 2013). The action of ADH oxidizes EtOH which results in the formation of ACD which is subsequently eliminated/metabolized by ALDH into acetate and eliminated from the body (for schematic depiction of EtOH metabolism see Figure 1). Alterations in either class of enzyme have been shown to produce alterations in ACD levels. An increase in the formation of ACD has been found to lead to an increase in the aversive symptoms (i.e., flushing, nausea, etc.) associated with EtOH consumption thereby decreasing further motivation to consume EtOH (Peng and Yin, 2009). Genetic studies have identified genetic polymorphisms in both ADH and ALDH which have been linked to a decreased susceptibility to develop an EtOH-use disorder (Edenberg, 2011). For instance, a recent study reported that Mexican Americans expressing the ADH1B^*^2 genotype were protected against EtOH-dependence (Ehlers et al., 2012). Such protection, against EtOH-dependence, likely occurs through a more rapid oxidation of EtOH resulting in significantly higher levels of peripheral ACD (Hurley and Edenberg, 2012). Research has indicated that an alteration in the expression of the ALDH2 gene results in a slower oxidation of ACD to acetate thereby resulting in a “Disulfiram-like” experience due to greater ACD levels (Ball, 2008). Recent endeavors have identified a polygenic contribution of the ADH gene cluster suggesting a potential role for several of the ADH genes in the development of alcoholism (Frank et al., 2012).

## Acetaldehyde formation within the CNS

Following the intake of EtOH, ACD is formed in the periphery, primarily by the activity of ADH in the liver. However, given high activity of aldehyde dehydrogenase (ALDH; the primary enzyme responsible for metabolizing ACD) within the blood brain barrier it was widely accepted that very little ACD could commute into the brain from the periphery (Hunt, 1996). Additional studies indicated that higher levels of ACD within the periphery may be capable of overwhelming peripheral ALDH, entering the brain within minutes (Ward et al., 1997; Quertemont et al., 2005). Metabolic activity resulting in the local formation of ACD within brain was not immediately clear and has been the topic of debate for several years. Research has indicated that ADH is not active within the brain and has established that EtOH is primarily metabolized through the activity of the catalase enzyme (Sippel, 1974; Zimatkin, 1991; Smith et al., 1997) and this enzyme remains relatively constant across different rat strains (Rhoads et al., 2012). *In vivo* studies support the activity of catalase as a key component in the formation of brain ACD following EtOH exposure as inhibition of catalase activity subsequently decreased brain ACD levels (Jamal et al., 2007). However, inhibition of catalase does not completely abolish ACD formation. Other metabolic pathways such as mitochondrial cytochrome P450 have also been found to produce ACD locally within the brain following the consumption of EtOH (Zimatkin et al., 1998; Zakhari, 2006). In mice, manipulation of cytochrome P450 has been found to alter overall sensitivity to EtOH (Vasiliou et al., 2006), EtOH consumption, and EtOH stimulated locomotor activity (Correa et al., 2009).

## Implication of ACD in the central actions of EtoH

Several studies that have made use of compounds that act to inhibit the formation of ACD or sequester ACD into a stable non-reactive adduct. Such experiments have implicated the local formation of ACD as an important aspect of the neurobiological and behavioral aspects of EtOH use/abuse. The compounds sodium azide and/or 3-amino-1,2,4-triazole (triazole) inhibit catalase activity, thereby decreasing ACD formation within the brain, and have been shown to alter EtOH related behaviors. For instance, both sodium azide and triazole significantly altered EtOH-induced locomotor activity when infused into the arcuate nucleus of the hypothalamus (Sanchis-Segura et al., 2005; Pastor and Aragon, 2008). Triazole has also been found to decrease the consumption of EtOH in both rats and mice (Aragon and Amit, 1992; Koechling and Amit, 1994), reduce EtOH induced motor depression in rats (Aragon et al., 1985) and EtOH induced locomotor activity in mice (Escarabajal et al., 2000). However, triazole has also been shown to cause a non-specific reduction in the consumption of saccharin-quinine solution (Rotzinger et al., 1994) and food intake (Tampier et al., 1995). Such data bring into question whether a reduction in EtOH consumption is a function of reduced ACD production or a general reduction in consummatory behavior caused by triazole. Recent studies have utilized a somewhat different approach to limiting the activity of the catalase system. The hydrogen peroxide (H_2_O_2_) scavenging compounds ebselen and alpha lipoic acid inhibit the formation of ACD through their reduction in the catalase-H_2_O_2_ reaction and subsequent formation of Compound I (Cohen et al., 1980). Ledesma and colleagues have demonstrated that exposure to both ebselen or alpha lipoic acid inhibit EtOH-stimulated locomotor activity in mice (Ledesma et al., 2012; Ledesma and Aragon, 2013).

Unlike compounds that directly affect brain catalase activity, thiol amino acid compounds, such as D-penicillamine or L-cysteine act to sequester ACD into a non-reactive stable adduct without altering EtOH metabolism (Cederbaum and Rubin, 1976; Nagasawa et al., 1978). Several studies have been conducted using these compounds which have added support for the role of ACD in the behavioral and pharmacological actions of EtOH. For instance, administration of either D-penicillamine or L-cysteine effectively reduced EtOH consumption and decreased EtOH conditioned place preference (CPP) in rats (Font et al., 2006b; Diana et al., 2008; Peana et al., 2008). Intra-cisterna magna injections of D-pennicillamine acted to block EtOH- and/or ACD appetitive conditioning to a surrogate nipple in newborn rats (March et al., 2013) and induced locomotor activity and tactile stimulus preference in preweanling rats (Pautassi et al., 2011). Mice exhibit a decrease in EtOH CPP and a reduction in EtOH-induced motor depression when treated with D-penicillamine (Font et al., 2005, 2006a). L-cysteine has been found to reduce nose-poke responding for ACD and EtOH during acquisition, maintenance, and reinstatement phases of testing (Peana et al., 2010, 2012) as well as inhibit EtOH and ACD induced CPP (Peana et al., 2009). Peripheral and central (intra-VTA) exposure to D-penicillamine significantly reduced expression of the alcohol-deprivation effect (ADE) as observed by a lack on an increase in EtOH consumption during the initial 3 post-abstinence measurements (Orrico et al., 2013). This finding offers support for the role of ACD in the expression of relapse-like behaviors as the ADE has been established as an animal model for EtOH relapse-drinking (for review see: McBride and Li, 1998).

Perhaps the most compelling evidence for the involvement of ACD in the central actions of EtOH has emerged from studies utilizing adenoviral and lentiviral vectors that alter catalase, ADH, or ALDH activity. Approximating the significantly higher activity of the ADH enzyme for individuals expressing the ADH1B^*^2 gene, mutated cDNA which encoded rADH-47His (the rat analogue for the ADH1B^*^2 gene) was peripherally administered to the University of Chile Bibulous (UChB) alcohol preferring rat line and resulted in significantly higher ACD blood levels while also significantly reducing EtOH consumption (Rivera-Meza et al., 2010, 2012). Similarly, an adenoviral vector coded for ALDH2 antisense RNA, to approximate clinical condition of reduced ALDH2 activity, produced comparable increases in blood ACD levels and decreases in EtOH consumption (Ocaranza et al., 2008; Rivera-Meza et al., 2012). Studies looking at the central administration of anticatalase (shRNA)- or ADH (rADH1)-encoding lentiviral vectors, which inihibit catalase synthesis or increases the activity of ADH respectively, have been found to alter EtOH-related behaviors. Administration of the anticalatase lentiviral vector into the ventral tegemental area (VTA) significantly reduced EtOH consumption and EtOH stimulated DA release in the AcbSh whereas the rADH1-encoding vector facilitated an increase in EtOH intake (Karahanian et al., 2011). Quintanilla et al. (2012) reported that insertion of an anti-catalase viral vector into the VTA resulted in the reduction of EtOH consumption when administered prior to EtOH testing. However, when the viral vector was administered during an ongoing EtOH drinking period, animals only exhibited a reduction in EtOH intake following a period of imposed abstinence during relapse-like drinking (Quintanilla et al., 2012). With regard to ADE expression, an additional study examining the effects of intra-VTA injection of anticatalase viral vector immediately following 67 consecutive days of EtOH exposure and immediately prior to a 15 day EtOH deprivation period, significantly reduced relapse drinking during both a first and second reinstatement of EtOH access (Tampier et al., 2013). Taken as a whole, research utilizing such cutting-edge techniques suggest that ACD possess a substantial role in the neurobiological actions of EtOH.

## ACD exhibits rewarding properties

While it is difficult to suggest that the behavioral and neurobiological effects of EtOH are completely dependent on the presence of ACD, there is a substantial amount of literature suggesting that ACD is involved to a significant extent. Studies examining the behavioral effects of ACD, with regard to EtOH reward, have reported that intra-cranial ventricular (ICV) administration of ACD acted to increase the consumption of and preference for EtOH in rodents (Brown et al., 1979, 1980; Amit and Smith, 1985) while peripheral administration of higher doses of ACD produced a conditioned taste aversion in several rat lines (Brown et al., 1978; Aragon et al., 1986; Kunin et al., 2000; Quintanilla et al., 2002; Escarabajal et al., 2003). Utilizing the UChB rat line (an alcohol preferring rat line), researchers have revealed that peripheral ACD exposure, at lower doses (50–100 mg/kg ACD) than those shown to produce a conditioned taste aversion (>200 mg/kg ACD), acted to significantly increase the consumption of EtOH over the two weeks following ACD administration (Tampier and Quintanilla, 2002). Taken together, such findings may suggest that ACD facilitates the development of tolerance to the aversive effects of EtOH thereby increasing EtOH consumption.

It has also been reported that ACD possesses rewarding/reinforcing properties itself as animals readily self-administered both ICV ACD (Amit et al., 1977; Brown et al., 1979, 1980) and intra-venous ACD (Myers et al., 1984a,b; Takayama and Uyeno, 1985). Central ICV administration of ACD produced a CPP (Smith et al., 1984). Extending on such findings, recent endeavors have reported that ACD, whether administered centrally or peripherally, produced a CPP in several rat lines (Quintanilla and Tampier, 2003; Peana et al., 2008; Spina et al., 2010). Adult rats peripherally treated with ACD exhibit a dose-dependent preference to a discrete olfactory stimulus (Quertemont and DeWitte, 2001). Rat pups exhibited a significant preference to an olfactory cue previously paired with ACD exposure (March et al., 2013) while pre-weanling rats exhibited an ACD-dependent stimulation of locomotor activity and tactile stimulus preference following EtOH administration (Nizhnikov et al., 2007; Pautassi et al., 2011). ACD has been shown to dose-dependently alter locomotor activity in adult animals as well. Rodent testing has reported that the central administration of lower doses of ACD resulted in significant increase in locomotor activty (Correa et al., 2003; Sanchez-Catalan et al., 2009) while higher doses, administered either centrally or peripherally, resulted in a significant depression of locomotor activity (Holtzman and Schneider, 1974; Ortiz et al., 1974; Myers et al., 1987; Durlach et al., 1988; Quertemont et al., 2004; Tambour et al., 2006). An early study also observed comparable biphasic effects utilizing a vapor exposure paradigm to deliver ACD (Ortiz et al., 1974). Recent studies have pursued the evaluation of the reinforcing effects of ACD via the oral route and reported that rats will actively nose-poke (Peana et al., 2010, 2012) or lever press to obtain ACD (Cacace et al., 2012). However, it is unlikely that the effects of oral ACD on the ACD self-administration were mediated via central ACD as Peana et al. (2010, 2012) reported that blood and brain ACD levels did not significantly differ between rats consuming oral ACD and those consuming water (Peana et al., 2010, 2012). Nonetheless, ACD possess rewarding properties itself which are related to (or underlie) the behavioral actions of EtOH.

## Acetaldehyde reactivity: byproducts of acetaldehyde

Acetaldehyde is a highly reactive compound that interacts with several endogenous neurochemicals in the brain to form a number of additional biologically active products (Cohen and Collins, 1970; Davis and Walsh, 1970; Walsh et al., 1970; Cohen, 1976). With regard to neurobiological and behavioral testing of the byproducts of ACD, the majority of attention has focused on two main classes of compounds which are formed through condensation of ACD with the catecholamines. The first class of compounds, the tetrahydroisoquinoline alkaloids (THIQs), are formed through both the direct and indirect condensation of ACD with the monoamines: dopamine, epinephrine, and norepinephrine (Cohen, 1976). The tetrahydro-beta-carbolines (TBCs) on the other hand, are formed through the reaction of ACD with the indoleamines: tryptophan and tryptamine (Buckholtz, 1980). The THIQs tetrahydropapaveroline (THP) and salsolinol (SAL) have received the most attention as to their role in alcohol use-disorders as both compounds can be detected in the brain following EtOH administration. The TBCs have received considerably less attention and contradictory data exists as to their contribution to the neurobiological effects of EtOH.

### Tetrahydropapaveroline

The formation of THP occurs via the condensation of dopaldehyde and dopamine. In this sense, ACD is indirectly associated with the formation of THP as ACD inhibits the breakdown/metabolization of dopaldehyde subsequently increasing THP levels in the brain (Davis and Walsh, 1970). Early studies observed an enhanced preference for EtOH and consumption of EtOH following ICV microinjections of low concentrations of THP in both rodents and primates (Melchior and Myers, 1977; Myers and Melchior, 1977; McCoy et al., 2003) while higher concentrations reduced both EtOH consumption and preference (Duncan and Deitrich, 1980). Manipulation of the mesolimbic DA pathway through microinjections of lower doses of THP into either the ventral tegmental area (VTA) or Nucleus Accumbens (Acb) accentuated EtOH preference in rats (Myers and Privette, 1989; Duncan and Fernando, 1991). Experiments were conducted in an effort to identify the neuroanatomical substrates of both the enhancing and aversive properties of THP with regard to alcohol related behaviors (Privette et al., 1988; Myers and Privette, 1989; Privette and Myers, 1989) however, research on the role of THP in EtOH-use disorders has slowed considerably over the past two decades.

### Salsolinol

Salsolinol (SAL; 1-methyl-6,7-dihydroxy-1,2,3,4-tetrahydroisoquinoline) is the most extensively studied byproduct of ACD in relation to EtOH-use disorders and studies aimed at examining the underlying contribution of SAL to the reinforcing properties of EtOH are still in full swing. The *in vivo* formation of SAL occurs primarily through non-enzymatic Pictet-Spengler condensation of DA with ACD (Lee et al., 2010) but has been hypothesized to occur through secondary processes as well (for review see: Hipolito et al., 2012). Several studies have sought to quantify SAL levels within the body and brain following EtOH ingestion with mixed results. Specifically in the rodent brain, studies have shown that EtOH exposure (via oral consumption or experimenter administered EtOH) increased (Rojkovicova et al., 2008) or did not alter (Lee et al., 2010) SAL levels in several brain regions. Nonetheless, there is a substantial amount of evidence suggesting that SAL is intricately involved with the rewarding properties of EtOH.

While the effect of SAL administration on EtOH intake received considerably less attention than that of ACD, early endeavors found that centrally ICV administered SAL caused animals to exhibit an increase in both their consumption of and preference for (Myers and Melchior, 1977; Duncan and Deitrich, 1980; Purvis et al., 1980). Altshuler and Shippenberg (1982) indicated that SAL possess similar discriminative properties compared to EtOH in that animals respond comparably when SAL is substituted for EtOH. More recently, several laboratories have shown that SAL exhibits reinforcing properties in the absence of EtOH. Animals exhibited a CPP for peripheral injections of 10 mg/kg SAL with higher (30 mg/kg) and lower (1 and 3 mg/kg) doses falling in a U-shaped dose response curve (Matsuzawa et al., 2000). Interestingly, when the animals were exposed to a conditioned fear stress (foot shock) the dose response curve shifted to the left (optimal dose: 3 mg/kg) as the animals exhibited a greater sensitivity to the reinforcing properties of SAL (Matsuzawa et al., 2000). Central administration of SAL (intra-VTA) has also been shown to induce a CPP in rats (Hipolito et al., 2011). Much like in response to EtOH, rats will exhibit a biphasic response in SAL-stimulated locomotor activity, specifically when SAL is microinjected into the VTA (Hipolito et al., 2010). Perhaps the most convincing evidence that SAL is reinforcing, even in the absence of EtOH, lies in data showing that animals will readily self-administer SAL into the posterior (p)VTA via intra-cranial self-administration at concentrations far below required to sustain the ICSA or EtOH or ACD (Rodd et al., 2008). Thus, research has outlined a clear role for SAL in the behavioral and neurobiological actions of EtOH and ongoing research is working toward the delineation of the nature of this contribution.

### Tetrahydro-betacarbolines

The role of TBCs in EtOH-use disorders has received considerably less attention than ACD and/or SAL. Findings have been somewhat inconsistent as early research indicated that peripheral injections of TBC derivatives reduced EtOH preference (Geller and Purdy, 1975). Central administration (ICV microinjections) of the TBC tryptoline had the opposite effect as it significantly increased both EtOH preference and EtOH consumption (Myers and Melchior, 1977; Tuomisto et al., 1982; Airaksinen et al., 1983; Huttunen and Myers, 1987; Adell and Myers, 1994). Co-administration THP and tryptoline resulted in a synergistic increase in EtOH preference and consumption (Myers and Oblinger, 1977). Hippocampal microinjections of TBCs produced alterations in both 5-HT and norepinephrine levels thereby significantly augmenting EtOH preference and consumption in low alcohol drinking (LAD) rats (Huttunen and Myers, 1987; Adell and Myers, 1995). Additionally, TBCs have been shown to possess an affinity for the delta opioid receptor (Airaksinen et al., 1984). Overall, however, the pharmacological properties of TBCs have yet to be fully examined.

## The neurobiological actions of EtoH and EtoH metabolites within the reward pathway

While early studies investigated the effects of the central and/or peripheral administration of ACD and/or SAL on the behavioral actions of EtOH (as discussed above), the dissemination of the underlying mechanisms of ACD and SAL at the neurobiological level has gained significant traction over the past three decades. The advent of a number of novel techniques (i.e., intracranial self-administration; ICSA) has allowed researchers to more thoroughly evaluate the neurobiological actions of drugs of abuse including EtOH and its metabolites. Research to date suggests that the neurobiological actions of EtOH and EtOH metabolites overlap to ultimately affect the development/expression of EtOH-use disorders. This section will present an overview of preclinical research focused on the neurobiological mechanisms within the brain reward pathway that have been identified to play a key role in the rewarding/reinforcing properties of EtOH, ACD, and the THIQs.

Numerous studies have implicated the mesocorticolimbic dopamine reward pathway (MCL) as a key mediator of the rewarding/reinforcing properties of virtually every major drug of abuse including EtOH (for review see: Di Chiara and Imperato, 1988). The MCL originates in the VTA and projects to several forebrain regions including the Acb (Oades and Halliday, 1987). An early study indicated that peripheral EtOH exposure stimulated DA neuronal activity within the substantia nigra (Mereu et al., 1984). A subsequent experiment found that peripheral injections of EtOH (0.5 mg/kg) significantly elevated DA levels within the AcbSh of freely moving rats (Di Chiara and Imperato, 1985). Subsequent research over the past 3 decades has elucidated a cascade of neurochemical events within the MCL that underlie EtOH reinforcement (for review see: Spanagel, 2009). For instance, it has been well documented that EtOH itself primarily targets N-methyl-D-aspartate (NMDA; Lovinger et al., 1989), 5-hydroxytryptamine 3 (5-HT_3_; Lovinger and Zhou, 1998), nicotinic acetylcholine (nAch; Narahashi et al., 1999), γ-aminobutyric acid A (GABA_A_) and glycine (Mihic et al., 1997; Mihic, 1999) receptors. The EtOH molecule also primarily interacts with non-ligand gated ion channels as EtOH inhibits L-type Ca^2+^ channels and opens G protein-activated inwardly rectifying K^+^ (GIRKs) channels (Vengeliene et al., 2008). Overall, such primary effects underlie and/or contribute to several secondary effects within the MCL (i.e., increases in DA efflux) that ultimately result in the rewarding/reinforcing properties of EtOH (Spanagel, 2009). Thus, research has established that the neurobiological actions of the EtOH molecule itself are important to the reinforcing properties of EtOH. However, given the dynamic nature of the neurobiological functioning of the MCL and the concurrent actions of EtOH metabolites, the extent to which the actions of the EtOH molecule itself contribute to overall EtOH reinforcement is somewhat tenuous.

**Figure 2:**
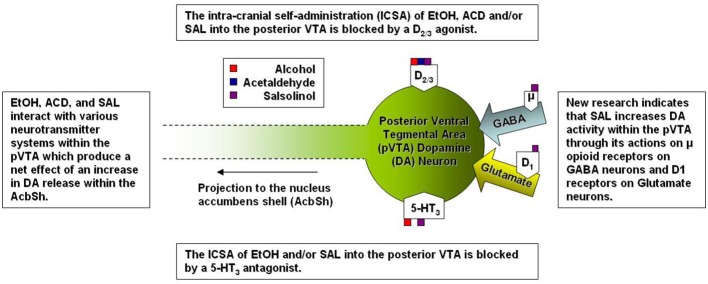
**A simplified representation of the sites of action for alcohol and alcohol metabolites on posterior ventral tegmental area dopamine neurons**.

Several studies have focused on the role of the projection from the VTA to the Acb in the neurobiological actions of EtOH as well as the metabolites of EtOH (see Figure 2). Specifically within the VTA, EtOH and ACD have been shown to activate DA neurons by significantly increasing their firing rate (Gessa et al., 1985; Brodie et al., 1990; Foddai et al., 2004), albeit through differing mechanisms (for review see: Deehan et al., 2013). Sequestering ACD formation through the direct infusion of D-penicillamine into the VTA inhibits DA neuronal activation by the intra-gastric administration of both EtOH and ACD (Enrico et al., 2009). Local application of an ADH (Foddai et al., 2004) or catalase inhibitor (Melis et al., 2007; Diana et al., 2008) in the VTA prevents EtOH stimulated increases in DA neuronal activity. These findings coupled with data from *in vitro* studies showing that ACD stimulates VTA DA neuronal activity at concentrations 1200–2000 fold lower than that required for EtOH suggest that ACD is critical component required for EtOH stimulated DA activity within the VTA (Brodie and Appel, 1998; Brodie et al., 1999; Diana et al., 2008). A recent paper has reported that SAL is also capable of stimulating VTA DA neuronal activity at concentrations 10-1,000 fold lower than the lowest effective concentration of ACD (Xie et al., 2012a).

Relative differences in effective concentrations between EtOH, ACD, and SAL have also been reported by studies examining the behavioral neuropharmacology of these compounds within the VTA. An early study indicated the alcohol preferring (P) rats (an animal model for alcoholism) would readily self-adminster EtOH directly into the VTA exhibiting a U-shaped dose response curve with 100 mg % being the most effective concentration (Gatto et al., 1994). Follow-up studies identified a regional heterogeneity within the VTA as both P and Wistar rats would self-administer EtOH into the posterior (p) VTA but not anterior (a) VTA at doses of 20-80 mM (Rodd et al., 2003, 2005). Much like EtOH, both ACD and SAL are self-administered into the pVTA in an inverted U-shaped dose response pattern and in congruence with neurophysiological data, the pVTA appears to be significantly more sensitive to the rewarding/motivational properties of each compound in a stepwise fashion (SAL > ACD > EtOH). For example, P rats self-administered ACD between the dose ranges of 6–90 μM (Rodd-Henricks et al., 2002; Rodd et al., 2005) whereas SAL ICSA was supported in a dose range of 0.03–0.3 μM (Rodd et al., 2008). For perspective, the optimal concentration for the ICSA of SAL is approximately 200-fold lower than the most effective concentration of ACD and 300 × 10^3^ lower than the optimal concentration of EtOH. Additionally, the co-infusion of the D_2/3_ agonist quinpirole (100 μM) blocked the ICSA of EtOH, ACD, and SAL into the pVTA (Rodd et al., 2005, 2008) suggesting that of DA neuronal activation within the pVTA is a common mechanism underlying the rewarding/motivational properties of EtOH, ACD, and SAL.

An alternative method to assess the efficacy of a given compound to stimulate DA neurons within the VTA involves microinjecting the compound into the VTA and measuring DA release in downstream projection structures (i.e., the Acb). An early study employed such a paradigm to examine the down-stream effects of microinjections of THP into the VTA on DA efflux within the core (AcbC) and the AcbSh reporting that a 13.6 μM microinfusion of THP increased DA efflux in the AcbC (94%) whereas the same dose decreased DA efflux in the AcbSh (51%; Myers and Robinson, 1999). Given that cannula placement were anterior to the VTA and the THP dose was well above the pharmacological range of the *in vivo* generation of THP (Haber et al., 1997; Baum et al., 1999), it is difficult to resolve whether THP altered DA neuronal activity directly or through a non-specific mechanism. Recent research, however, has utilized similar equipment as that employed for ICSA experiments to examine the effects of intra-pVTA microinjections of EtOH, ACD, and/or SAL on DA levels downstream within the AcbSh (Ding et al., 2009, 2011; Deehan et al., 2013). Ding et al. (2009) reported that pulse microinjections of 200 mg% (~44 mM) EtOH was the most efficacious dose at stimulating DA efflux in the AcbSh of Wistar rats. Utilizing the same range of doses of ACD and SAL that were reliably self-administered via ICSA (Rodd et al., 2005, 2008), Deehan et al. (2013) reported that Wistar rats exhibited comparable U-shaped dose response curves for DA efflux in the AcbSh following pulse microinjections of ACD and/or SAL into the pVTA. Along the same lines as previous observations utilizing alternative paradigms, DA neurons within the pVTA exhibited a significantly greater sensitivity to ACD and/or SAL compared to EtOH. Pulse microinjections of 23 μM ACD or 0.3 μM SAL were effective at significantly increasing DA efflux within the AcbSh to levels 200 and 300% above baseline respectively (Deehan et al., 2013). Moreover, this was observed for an ACD dose that was over 1800 fold, and a SAL dose that was 147,000 fold lower, than the peak dose of EtOH. These data further suggest that the pVTA is differentially sensitive to EtOH, ACD, and SAL in a manner that is consistent with the production of ACD and SAL through conventional metabolic processes.

The findings from the microinjection/microdialysis study by Deehan et al. (2013) extend on previous research that reported increases in accumbal DA in response to local exposure of higher concentrations of ACD or SAL within the pVTA. The reverse microdialysis of 75 μM ACD in the pVTA stimulated DA release in the AcbSh to 150% of baseline (Melis et al., 2007; Diana et al., 2008) while Hipolito et al. (2011) reported that a microinjection of SAL (150 μM) within the pVTA caused an increase in AcbSh DA to 130% of baseline. However, SAL has been shown to modulate DA levels within the AcbC and AcbSh in an opposing manner. Local perfusion of SAL via reverse microdialysis, over the course of a 20-min sample, significantly increased DA levels in the AcbC but decreased DA levels in the AcbSh (Hipolito et al., 2009) in a manner consistent with the effects of selective μ- and δ-opioid receptor agonists reported by the same lab (Hipolito et al., 2008). Although the lowest concentration of SAL (5 μM) used by Hipolito et al. (2009) was significantly higher than the optimal concentration (0.3 μM) that stimulated activity in DA neurons within the pVTA (Deehan et al., 2013), ICSA studies have shown that the AcbSh is significantly less sensitive to the rewarding properties of SAL with the greatest level of responding exhibited for the 3.0 μM concentration of SAL (Rodd et al., 2003). Additionally, the rewarding properties of SAL within the AcbSh were found to be dependent on post-synaptic activation of DA receptors as the D_2/3_ antagonist (sulpiride) completely abolished ICSA responding for SAL.

Overall, there are several neurobiological mechanisms that underlie the EtOH, ACD, or SAL induced stimulation of DA neuronal activity within the pVTA, not all of which participate equally across the three compounds. For instance, research has implicated 5-HT_3_ receptors in the reinforcing properties of EtOH and SAL but not ACD. The compound ICS 250,390 (a 5-HT_3_ receptor antagonist) selectively prevents the ICSA of both EtOH and SAL but does not affect the ICSA of ACD (Rodd et al., 2005, 2008). This stands to reason as EtOH possesses an affinity for 5-HT_3_ receptors (Lovinger and White, 1991) and SAL increases the efflux of 5-HT within the rat striatum (Maruyama et al., 1993) but ACD does not exhibit an affinity for 5HT_3_ receptors (Li, 2000). Within the striatum SAL decreases the metabolization of 5-HT through a reduction in metabolizing enzymes resulting in an increase in 5-HT levels to 20 times that of DA (Nakahara et al., 1994). Similar findings have been reported with regard to DA as SAL increases catecholamine levels within the brain through a combination of the inhibition of reuptake (Heikkila et al., 1971; Tuomisto and Tuomisto, 1973; Alpers et al., 1975) and a reduction in the metabolizing enzymes such as catecholmethyltransferase and monoamine oxidase (Collins et al., 1973; Alpers et al., 1975).

From early on, studies had outlined a substantial role for the mu opioid receptor (MOR) in the neurobiological actions of EtOH, ACD, and SAL within the MCL. Naltrexone, a general opioid antagonist with an affinity for all three opioid receptors (mu, delta and kappa), has been approved by the FDA for use in the treatment of alcohol use disorders (Johnson and Ait-Daoud, 2000). Preclinical data indicate that naltrexone decreases both free-choice consumption and the operant self-administration of EtOH (for review see: Gianoulakis, 2009). For instance, EtOH has been found to directly alter the release of opioid peptides (Jarjour et al., 2009) and both naltrexone and β-funaltrexamine (β-FNA; a selective MOR antagonist) reduced the duration of DA release within the AcbSh caused by intra-VTA microinjections of EtOH (Valenta et al., 2013). Microinjections of higher concentrations of EtOH into the VTA stimulate locomotor activity that is prevented by the co-administration of β-FNA (Sanchez-Catalan et al., 2009) or the co-administration of D-penicillamine (Marti-Prats et al., 2010) suggesting that the locomotor activating effects of EtOH within the VTA require MOR activation as well as the presence of ACD.

Both ACD and SAL possess locomotor stimulating properties within the VTA and much like EtOH, the activation of locomotor activity has been reported to be dependent on MOR activation (Sanchez-Catalan et al., 2009; Hipolito et al., 2011). To date, there is a lack of research investigating the effects of MOR manipulation on the self-administration of ACD or SAL. An early study observed a decrease in the IV self-administration of ACD when animals were treated with naloxone (Myers et al., 1984a). Further, the oral self-administration (nose poke responding) of ACD was decreased by naltrexone and naloxonazine (a selective MOR_1_ antagonist; Peana et al., 2011). Naltrexone also acted decrease extracellular signal-regulated kinase phosphorylation within the Acb caused by ACD self-administration (Peana et al., 2011). However, the full transgression from MOR activity to increased DA neuronal activity within the pVTA, and subsequent increase in DA release downstream, is indeed complex. Xie and colleagues have reported that SAL stimulates DA neurons within the pVTA indirectly by activating MORs which in turn inhibit of gama-amino butyric acid (GABA) neurons (Xie et al., 2012a) while also increasing glutamatergic signaling into the pVTA (Xie and Ye, 2012b). Overall, it is likely that the rewarding/reinforcing properties of both ACD and SAL in the pVTA are dependent on DA release within the AcbSh and the DA activity is modulated via MORs. After all it has been shown that direct stimulation of MORs increase DA release within the AcbSh (Spanagel et al., 1992) an similar effect to that observed following microinjections of EtOH, ACD, or SAL into the pVTA (Ding et al., 2009; Deehan et al., 2013). Although there are no studies focused on the role of MOR activity in the central self-administration of ACD and/or SAL, the current body of literature has implicated MOR activity within the pVTA as a key mediator of the neurobiological action of ACD and/or SAL on DA neurons. Future research will help to further elucidate other contributory structures and neurochemical systems, within the MCL, with regard to ACD and SAL.

## General summary

The action of EtOH within the CNS is extremely complex yet the current body of literature has outlined a significant role for ACD and SAL in the modulation of the behavioral and neurological effects of EtOH. It has been shown that EtOH can act directly within the VTA to stimulate DA neurons (Gessa et al., 1985; Lovinger and White, 1991; Brodie et al., 1999; Ye et al., 2001) and accumulating evidence suggests that both ACD and SAL exhibit distinct actions on neurobiological processes within the MCL. The utilization of inhibitory/sequestering agents preventing the conversion from EtOH into ACD clearly affect EtOH consumption and reinforcement further supporting the role for the metabolites of EtOH in EtOH-use disorders. Thus, convergent evidence supports the following assertions: (1) within the CNS, EtOH is capable of altering neurobiological and behavioral processes, (2) evidence exists supporting the notion that the actions of EtOH, are, in part, mediated by the metabolites ACD and SAL formed during metabolic processes, (3) both ACD and SAL possess reinforcing properties within the MCL at levels shown to be pharmacologically relevant, and (4) further research focused on examining the central effects of EtOH and EtOH metabolites will greatly improve our understanding of how these compounds function in regard to the development/expression of EtOH-use disorders. Overall, the manifestation of EtOH-use disorders in the clinical population is undoubtedly a result of a complex and interrelated series of central and peripheral effects of EtOH and the metabolites of EtOH. Research aimed at increasing our understanding of such a complex system will facilitate the development of successful pharmaocterapeutic treatments for individuals suffering from, or are at a high risk to develop, an EtOH-use disorder.

### Conflict of interest statement

The authors declare that the research was conducted in the absence of any commercial or financial relationships that could be construed as a potential conflict of interest.

## Abbreviations

Triazole: 3-amino-1,2,4-triazole
ACD: Acetaldehyde
ADH: Alcohol Dehydrogenase
ALDH: Aldehyde Dehydrogenase
CNS: Central Nervous System
CPP: Conditioned Place Preference
D_2_: Dopamine 2
DA: Dopamine
EtOH: Ethanol
GABA: Gamma-aminobutyric Acid
GLU: Glutamate
ICV: Intra-Cerebral Ventricular
IV: Intra-venous
μM: Micro-molar
mM: Milli-molar
MOR: Mu Opioid Receptor
AcbC: Nucleus Accumbens Core
AcbSh: Nucleus Accumbens Shell
Acb: Nucleus Accumbens
5HT_3_: Serotonin 3
5-HT: Serotonin
TBCs: Tetrahydrobetacarbolines
SAL: Salsolinol
THIQs: Tetrahydroisoquinoline alkaloids
THP: Tetrahydropapaveraoline
FDA: United States Food and Drug Administration
VTA: Ventral Tegmental Area.
